# Astragalus Tuberculosis: A Case Report and Review of the Literature

**DOI:** 10.7759/cureus.1708

**Published:** 2017-09-22

**Authors:** Yugal Karkhur, Vivek Tiwari, Jeetendra Lodhi, Anurag Tiwari

**Affiliations:** 1 Department of Orthopaedics, Maulana Azad Medical College, New Delhi, India; 2 Department of Orthopaedics, All India Institute of Medical Sciences, New Delhi, India

**Keywords:** talus, tuberculosis, tb, bone tuberculosis

## Abstract

Osteo-articular tuberculosis continues to be a major global pandemic, with its greatest impact in the third-world countries. Among osteo-articular tuberculosis, plantar localisation, particularly isolated involvement of the talus is an extremely rare event. We discuss the case of a 20-year-old male diagnosed with isolated tuberculosis of right talus without the radiological involvement of the distal tibia, fibula or calcaneum. The diagnosis was made with the help of magnetic resonance imaging and confirmed through core biopsy of the talus. He was treated with multi-drug antitubercular chemotherapy and ankle immobilization with protected weight bearing with good results.

## Introduction

Osteo-articular tuberculosis (TB) has remained a diagnostic challenge, particularly when the disease affects unusual sites. Among the different bony sites known to be affected by TB, the involvement of the foot is a relatively unusual event [1]. The diagnosis of foot TB is challenging due to vague symptomatology and a presentation which closely resembles that of other more common disorders affecting this region [2]. Delaying the accurate diagnosis in such cases can translate in spreading the disease to the adjacent joints thereby causing widespread destruction. Isolated TB of the talus (Astragalus) bone is very rarely encountered. There have been less than fifteen cases of talus TB described in the literature to date [1-2]. We present the case of a young male suffering from isolated TB of right talus treated conservatively with anti-tubercular chemotherapy and immobilization of the ankle joint with protected weight-bearing leading to a good functional outcome.

## Case presentation

**Figure 1:**
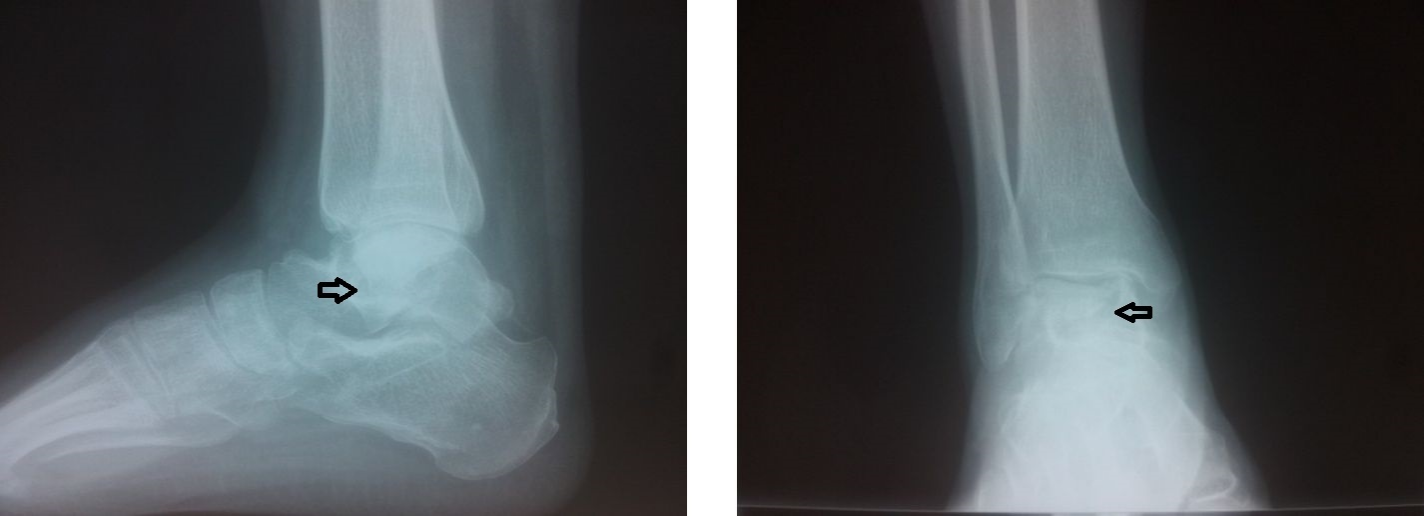
Plain radiographs of the right ankle's anteroposterior and lateral views. Extensive lytic lesion in the body of talus (black arrows).

**Figure 2:**
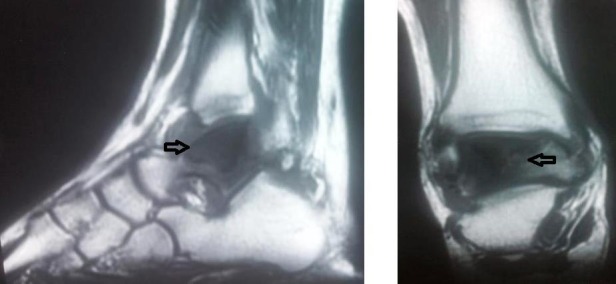
Magnetic resonance imaging (MRI) of the right ankle. Hypo-intense lesions in the talus bone on T1 weighted images (black arrows), consistent with an inflammatory focus in the talar bone.

A 20-year-old male patient presented to the orthopedics outpatient department with complaints of pain and swelling in the right ankle for three months. He also complained of difficulty in walking during the last one week. There was no history of fever, weight-loss, loss of appetite, trauma or similar complaint in the other foot. His general physical examination was unremarkable. On local examination, there was tenderness in his right ankle joint with the painful and restricted range of motion. Laboratory findings demonstrated an elevated erythrocyte sedimentation rate (ESR) of 76 mm per hour (Westergren method), a positive C-reactive protein (CRP) test and white blood cell (WBC) count of 10,000 cells per microlitre- 65% neutrophils, 27% lymphocytes, 6% monocytes, 1% basophils and 1% eosinophils. The Mantoux skin test was found to be negative. Plain radiographs of the chest were normal. The X-ray of the ankle obtained in two orthogonal views: anteroposterior (AP) and lateral demonstrated a widespread irregular lytic lesion in the talus without the associated involvement of the adjacent bones (Figure 1). The magnetic resonance imaging (MRI) of the right ankle showed areas of low and high signal intensity in talus on T1 and T2 weighted images, respectively, without the involvement of the ankle mortise (Figure 2). After informed consent from the patient, core biopsy was done from the lesion under local anesthesia and under radiological guidance. Upon histo-pathological microscopic examination, the biopsy sample revealed granuloma with central caseating necrosis. Subsequently, tubercular infection in talus was tested by acid-fast staining and polymerase chain reaction (PCR) examination for TB bacilli, both of which were found to be positive in the obtained tissue sample.

**Figure 3:**
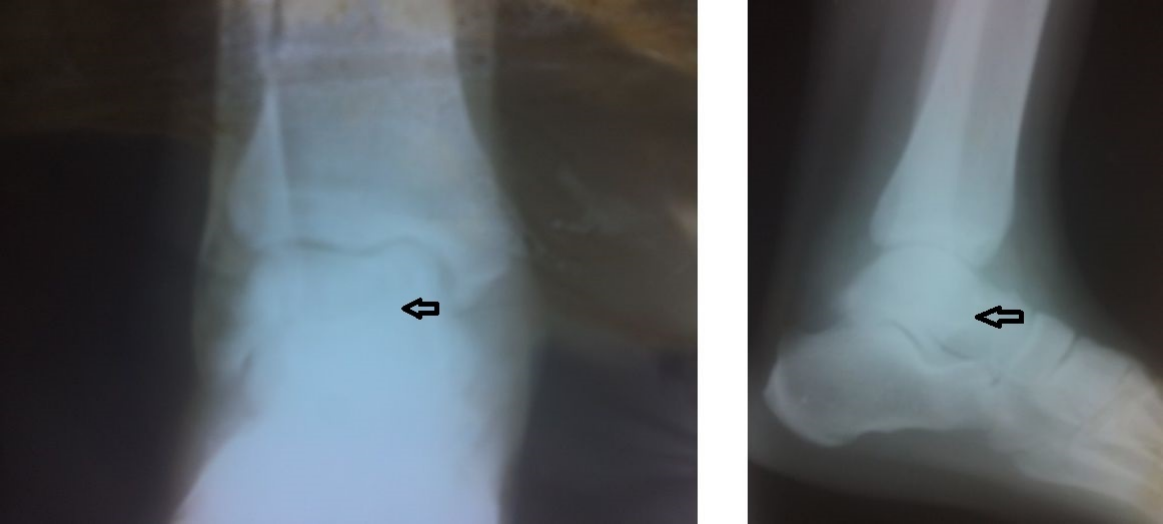
Follow-up plain radiographs of the right ankle's anteroposterior and lateral views. Healed lesion in the talus (black arrows) after 12 months of anti-tubercular chemotherapy.

Anti-TB chemotherapy was administered to the patient for 12 months, which included four drugs (isoniazid (INH), Pyrazinamide, Ethambutol, and Rifampicin) for the first three months followed by two drugs (INH and Rifampicin) for the subsequent nine months. A non-weight bearing was kept on the affected limb and the ankle joint was fully immobilized in the plaster of Paris (POP) cast during the first two months. Partial-weight bearing mobilization was initiated after two months. The patient obtained a painless ankle range of motion with full weight-bearing mobilization after finishing the 12 months chemotherapy. After 12 months, plain radiographs of the right ankle anteroposterior and lateral views demonstrated bone healing in the talus (Figure 3).

## Discussion

Tuberculosis (TB) infection is a grave problem for the medical society, especially in the third-world healthcare systems. Although it manifests in the pulmonary system, in the majority of cases, non-pulmonary systems including musculoskeletal system can get involved in as much as for 30% of the patients [3]. The most common sites affected osteoarticularlar TB include the spine, hip, and knee [4]. Involvement of the foot and ankle region by this unforgiving disease is relatively less common with the calcaneum being the most frequent bone affected [5]. Furthermore, the isolated tubercular disease of the talar bone is extremely rarely described the in literature.

Haraldsson reported a case of talus TB in a two-and-half-year-old child following administration of Bacillus Calmette–Guérin (BCG) vaccine [6]. Due to lack of awareness and clinical suspicion owing to its rarity, there was a delay of five weeks in the correct diagnosis of isolated talus TB in a six-year-old child [7]. Goksan, et al. reported conservative treatment with a combination of three drug chemotherapy for a case of tuberculous osteitis of the talus in a nine-year-old child [8]. Jockheck, et al. treated a Vietnamese patient with tubercular osteitis of the talus with partial astragalectomy along with tibio-calcaneal fusion using the Charnley fixator [2]. In another report of isolated talus TB, Boussouga, et al. treated a 66-year-old patient conservatively with three drug anti-TB chemotherapy [1]. In the above case, standard laboratory investigations, as well as imaging studies like MRI and X-ray, failed to establish the diagnosis of TB, which was done by histopathological examination.

Anand and Sood reported operative treatment with curettage and bone grafting in a case of the osteolytic type of talar TB in an eight-year-old child with unremarkable routine blood investigations, erythrocyte sedimentation rate (ESR) and Mantoux test [9]. In a retrospective analysis of the foot and ankle TB cases, Dhillon, et al. found only a single case of talus TB out of 992-foot cases over a period of 20 years [5]. Dahuja, et al. reported a case of isolated TB of the talus treated with surgical curettage and debridement along with anti-TB chemotherapy giving good results [3]. Halwai, et al. reported the use of transmalleolar osteotomy for surgical debridement and obtaining tissue samples in a talar TB case who had a normal ankle joint range of motion after six years [10].

Owing to the unusual clinical presentation and inconclusive routine investigations in most of the cases of talus TB, such patients suffer from a delay in diagnosis leading to increased morbidity. Awareness about these rare varieties of osteoarticular TB can help orthopedic surgeons in keeping them as differentials in all the inflammatory pathologies of foot and ankle. Our case also highlights the importance of MRI and histopathological evaluation in confirming the correct diagnosis. After early diagnosis, most of such patients can be treated conservatively with adequate doses of anti-tubercular chemotherapy for the appropriate duration. The chemotherapy needs to be administered for a longer duration in bone TB cases as compared to pulmonary TB. The surgical treatment in the form of debridement, curettage or talectomy followed by tibiocalcaneal arthrodesis may be required in unresponsive and incalcitrant cases depending on the severity of talar destruction.

## Conclusions

A high index of clinical suspicion together with positive imaging features on MRI, staining for acid-fast bacilli and histopathological examination of the tissue samples could help us in arriving at the prompt diagnosis of this rare case of isolated talus TB. Early diagnosis prevented disease progression to the adjacent bones and joints, thereby reducing the amount of the tissue destruction. Conservative management with long-duration multidrug, anti-TB chemotherapy along with immobilization helped in complete resolution of the infection with good functional results. The knowledge and awareness about the occult presentation of these cases will help surgeons in early diagnosis and timely intervention, thus providing good functional results.
